# Supplementary material for: A Low-Protein High-Fat Diet Leads to Loss of Body Weight and White Adipose Tissue Weight via Enhancing Energy Expenditure in Mice
Source: Metabolites. 2021 May 11;11(5):301. doi: 10.3390/metabo11050301 (PMC8150844; doi:10.3390/metabo11050301)
Supplement: Supplementary file 1 [file metabolites-11-00301-s001.zip › metabolites-1203768-supplementary.pdf]

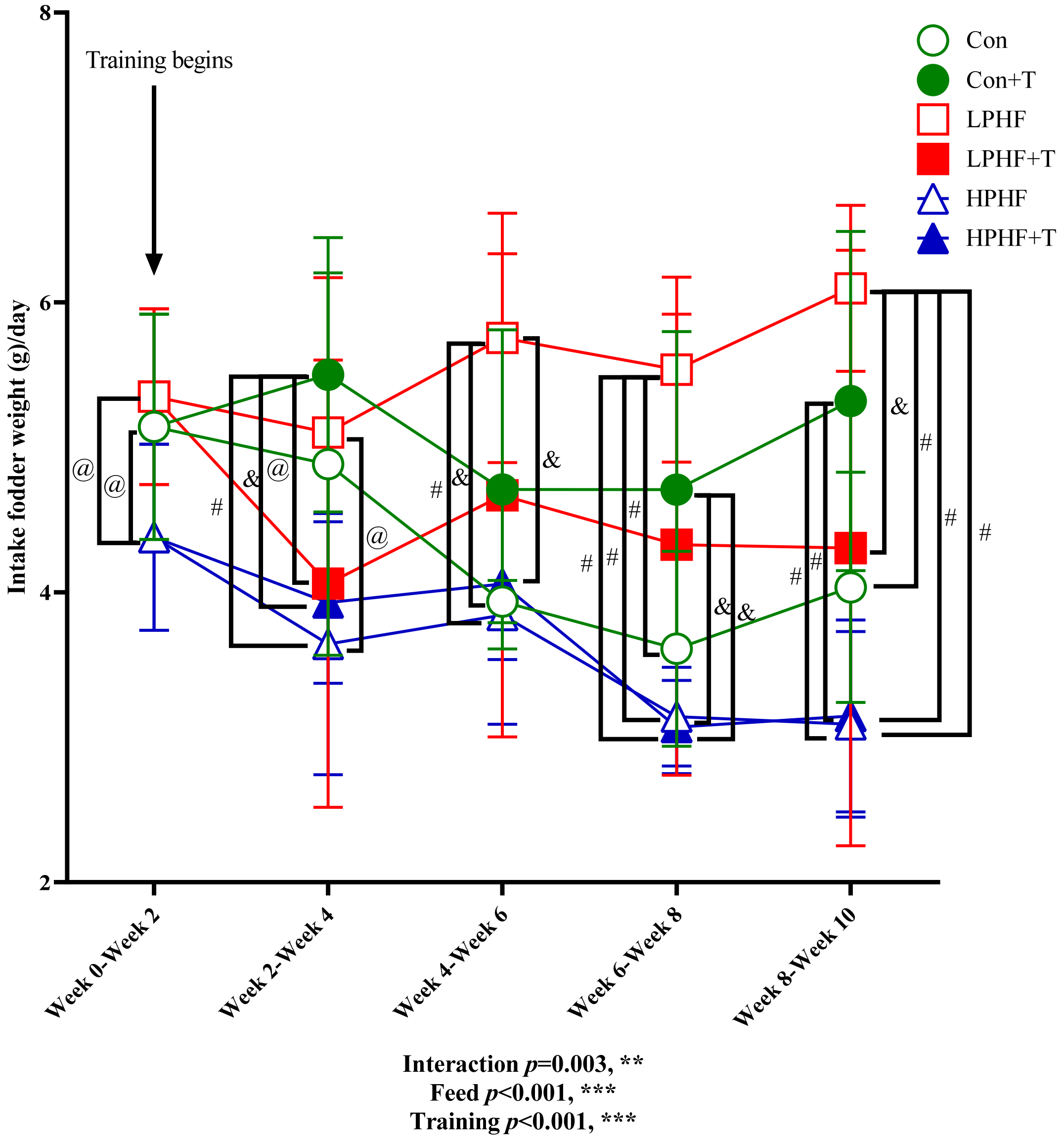

Figure S1. Changes of intake fodder weight throughout the experiment. Con, Con+T, LPHF, LPHF+T, HPHF, and HPHF+T represent chow diet, chow diet plus training, low-protein high-fat diet, low-protein high-fat diet plus training, high-protein high-fat diet, and high-protein high-fat diet plus training, respectively. Feed, training, and interaction indicate a dominant effect of diet, treadmill exercise, and the interactive effect between feed and training, respectively. ** $p < 0.01$; *** $p < 0.001$. @, $p < 0.05$; &, $p < 0.01$; #, $p < 0.001$. Data are presented as the mean ± SD. n = 8 for each group. Significance of differences between means was determined by Tukey's post hoc test when ANOVA revealed a significant effect from interaction of diet and training.

Table S1. Effects of LPHF, HPHF and/or training on absolute tissues/organs weight.

Tissue/Organ	Con groups		LPHF groups		HPHF groups		Significance		
	**Con**	**Con+T**	**LPHF**	**LPHF+T**	**HPHF**	**HPHF+T**	**Training**	**Feed**	**Interaction**
WAT, g	0.90 ± 0.31[e]	0.43 ± 0.18[ef]	0.53 ± 0.11[e]	0.45 ± 0.12[ef]	1.93 ± 0.72[abcdf]	1.01 ± 0.37[bde]	***	***	**
BAT, g	0.11 ± 0.03	0.10 ± 0.03	0.09 ± 0.02	0.20 ± 0.36	0.13 ± 0.05	0.08 ± 0.03	n.s.	n.s.	n.s.
SKM, g	0.18 ± 0.02	0.18 ± 0.01	0.14 ± 0.01	0.14 ± 0.01	0.18 ± 0.01	0.18 ± 0.01	n.s.	***	n.s.
Liver, g	1.11 ± 0.14	1.10 ± 0.09	0.98 ± 0.12	0.98 ± 0.06	1.30 ± 0.31	1.20 ± 0.10	n.s.	***	n.s.
Kidney, g	0.32 ± 0.03	0.33 ± 0.03	0.23 ± 0.02	0.24 ± 0.01	0.34 ± 0.04	0.36 ± 0.02	n.s.	***	n.s.

WAT, BAT, and SKM represent white adipose tissue; brown adipose tissue; and skeletal muscle, respectively. Con, Con+T, LPHF, LPHF+T, HPHF, and HPHF+T represent chow diet, chow diet plus training, low-protein high-fat diet, low-protein high-fat diet plus training, high-protein high-fat diet, and high-protein high-fat diet plus training, respectively. Feed, training, and interaction indicate a dominant effect of diet, treadmill exercise, and the interactive effect between feed and training, respectively. n.s., no significance was observed. ** $p < 0.01$ and *** $p < 0.001$. Data are presented as the mean ± SD. n = 8 for each group. Significance of differences between means was determined by Tukey's post hoc test when ANOVA revealed a significant effect from interaction of diet and training. [a-f], significantly different from Con, Con+T, LPHF, LPHF+T, HPHF, and HPHF+T, respectively.
